# Revealing the complex role of *CDKL5* in developmental epilepsy through a calcium channel related vision

**DOI:** 10.1186/s42494-024-00162-7

**Published:** 2024-05-03

**Authors:** Mengqi Yan, Xiongfeng Guo, Cenglin Xu

**Affiliations:** Key Laboratory of Neuropharmacology and Translational Medicine of Zhejiang Province, School of Pharmaceutical Sciences, The Second Affiliated Hospital of Zhejiang Chinese Medical University (Zhejiang Xinhua Hospital), Zhejiang Chinese Medical University, Hangzhou, 310053 China

**Keywords:** Developmental and epileptic encephalopathies, CDKL5, CDKL5 deficiency disorder

## Abstract

Developmental and epileptic encephalopathies are severe neurological conditions in clinical practice, among which loss-of-function mutations in brain-enriched serine-threonine kinase cyclin dependent kinase like-5 (CDKL5) exists as one of the most common types. It is unknown, therefore, how precisely *CDKL5* mutations lead to neuronal hyper-excitation. A recent study that looked at the connection between voltage-gated calcium channel Cav2.3 and CDKL5 in an experimental context was published in Nature Communications. This study has revealed that Cav2.3, a physiological phosphorylation target of CDKL5, would show delayed inactivation and increased cholinergic stimulation in *CDKL5* knock out conditions. This would in turn cause neuronal hyperexcitability and related enhanced seizure susceptibility. This work, in our opinion, provided fresh insight into the epileptic encephalopathies linked to CDKL5 and highlighted Cav2.3 as a possible target for it.

## Background

Developmental and epileptic encephalopathies (DEE), characterized by severe seizure activities arising in childhood as well as cognitive impairment during the developmental period, are not rare for neurologists [1]. Although the susceptible genes of more than 90 DEEs have been recognized with the development of medical genetics diagnosis [1], a substantial challenge still exists: the exact molecular mechanisms from gene mutations to epileptic phenotypes. For brain-enriched serine-threonine kinase cyclin dependent kinase like-5 (CDKL5), cumulative evidence suggests that de novo loss-of-function (LOF) mutations would lead to CDKL5 deficiency disorder (CDD) — one of the most common types of DEEs with infantile-onset and intractable seizures [2, 3]. Nevertheless, the precise processes by which CDKL5 LOF would result in neuronal hyperexcitability are still unclear.

## Main text

**Fig. 1:**
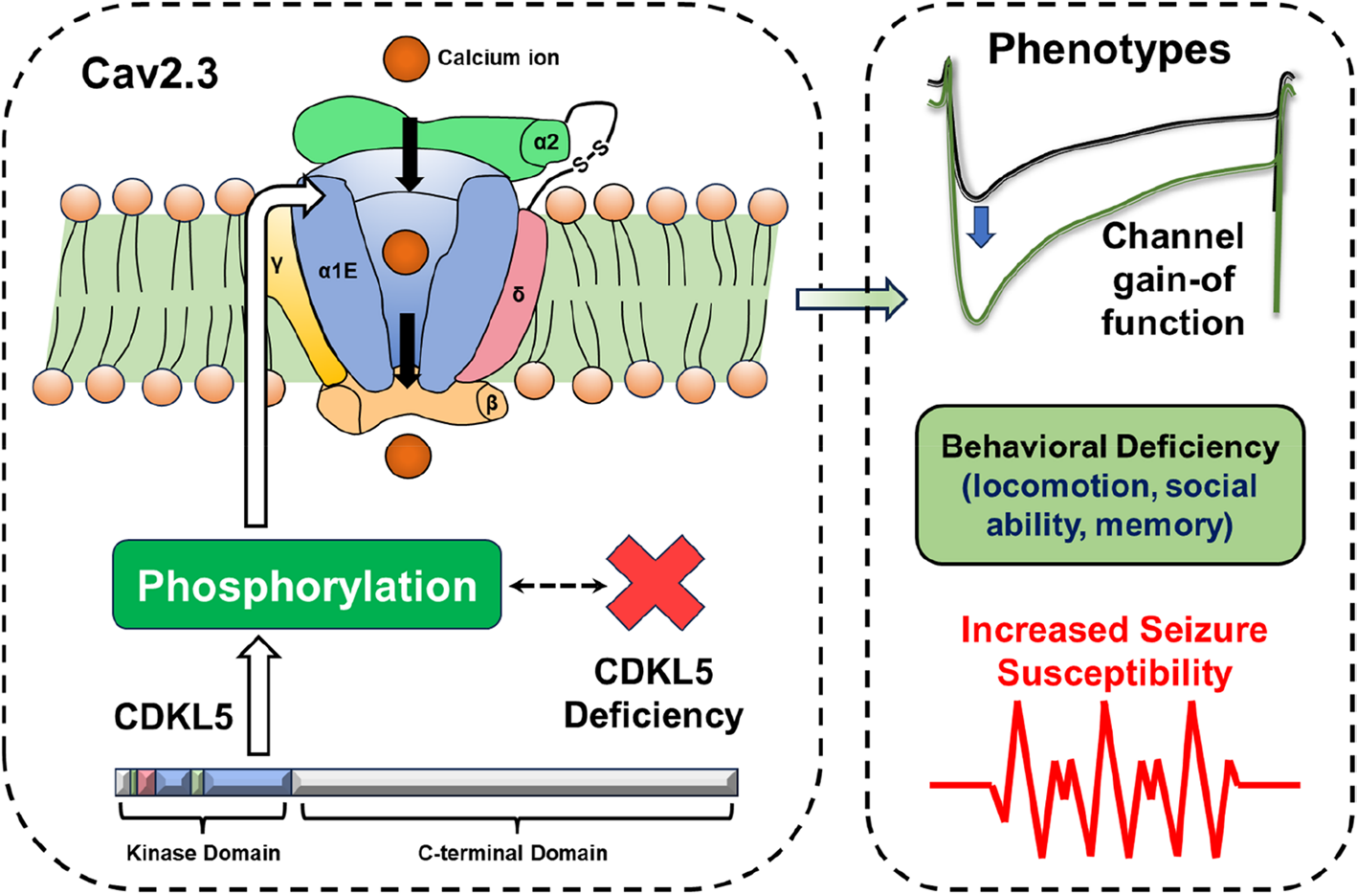
CDKL5 deficiency leads to CDD phenotypes through influencing the substrate Cav2.3 functions (modified from Sampedro-Castaneda et al [4)]. Cav2.3 contains the α1E channel pore subunit, which contains CDKL5 phosphorylation site at S15/14, along with associated proteins Cavβ and α2δ. The deficiency of CDKL5 would cause the gain-of-function of Cav2.3, leading to CDD-like behavioral deficiency and increased seizure susceptibility as a consequence

Recently, researchers from the Francis Crick Institute of UK published an intriguing study in *Nature Communications* [4]. It first revealed that the hyperexcitability associated with CDD is closely linked to the unbalanced function of the voltage-gated calcium channel Cav2.3 (Fig. 1), which provides new insight into the disease and the search for possible therapeutic targets.

In this study, the authors performed isotope labelling of amino acids in cell culture to differentially identify newly synthesized proteins in primary cortical cultures from both wild type and *CDKL5*^*−/−*^ mice. By comparing the protein phosphorylation levels, they unexpectedly found that the ion channel Cav2.3 showed a strikingly reduced phosphorylation at S15 (which matches the CDKL5 RPXS/T* consensus motif) in *CDKL5/*^*−/−*^ neurons. To further examine whether *CDKL5* would phosphorylate Cav2.3 S15/S14, the authors generated a phosphospecific antibody. According to their findings, co-overexpressing Cav2.3 and the CDKL5 kinase domain resulted in strong Cav2.3 phosphorylation in vitro, while either overexpressing phosphomutant Cav2.3 or kinase-dead CDKL5 mutant would not lead to similar findings. This phenomenon was further validated, as the authors claimed that in *CDKL5*^*−/−*^ mice, Cav2.3 phosphorylation was consistently reduced. Moreover, phosphorylation at Ser15 (pS15) of Cav2.3 was decreased in iPSC-derived neurons obtained from CDD patients.

Then, the authors aimed to test the hypothesis that the decreased phosphorylation at Ser14 (pS14) of Cav2.3 caused by *CDKL5*^*−/−*^ would lead to altered channel properties and neuronal excitation. They designed an in vitro cell platform by expressing the human auxiliary subunits β3 and α2δ1 onto the HEK293 cell line. The cell line was also transiently transfected with human α1E (containing serine 15) and full-length CDKL5 plasmids. Through performing whole-cell recordings, the Cav2.3-mediated currents revealed slower decay kinetics in S14A mutant channels, while this difference could not be seen in the absence of CDKL5. Notably, S14A Cav2.3 showed a significant increase in current density at maximum activation voltages compared to WT Cav2.3. Given that different β subunits may influence the biophysical properties of calcium channel [5], parallel experiments were performed in a HEK293 cell line stably expressing human α2δ1 and β1b, yielding similar findings to the β3 subunits. Due to the link of Cav2.3 and its upstream muscarinic acetylcholine receptors [6], the authors investigated whether muscarinic receptor enhancement would also be affected by CDKL5 phosphorylation. A co-expressing strategy involving Cav2.3 and its main upstream muscarinic receptor type 3 or 1 (M3 or M1), as well as CDKL5 was performed, the co-transfection of the four plasmids were confirmed by immunostaining. Cav2.3 exhibited a shift of V_1/2_ toward a hyperpolarizing direction caused by the cholinergic agonist carbachol. Interestingly, this shift was greater without Ser14 phosphorylation, implying an additional gain-of-function due to loss of CDKL5. Additionally, the authors excluded the possibility that Gβγ may influence the pS14-dependent gain-of-function. Thus, the authors claimed that CDKL5-mediated pS14 could speed up the inactivation of Cav2.3, and the absence of CDKL5 resulted in the gain of function of Cav2.3.

The authors then attempted to confirm those findings in neurons by using CRISPR-Cas9 genome editing to generate Cav2.3 S15A phosphomutant mice. The authors reported that the total S15 phosphorylation levels were < 10% of control levels in homozygous S15A mice (HOM S15A), while the number was about 50% in heterozygous ones. The findings of the HEK293 cell line were confirmed by the authors using whole-cell recordings in hippocampus slices from HOM S15A mice, which revealed a substantially slower inactivation of isolated Ca^2^^+^ currents. Next, the impact of Cav2.3 S15A on neuronal excitability was examined. When injected with high depolarized currents, the S15A neurons responded with fewer spikes and showed depolarization block towards the end of the stimulus. More significantly, the percentage of neurons exhibiting sustained depolarizing plateau potentials or afterdepolarization elicited by Carbachol was higher in HOM S15A mice. Taken together, Cav2.3 phosphomutant mice showed increased neuronal depolarization due to the facilitation of Cav2.3 gating without S15 phosphorylation upon the cholinergic agonists application.

Lastly, the researchers attempted to investigate the behavioral and EEG abnormalities in HOM S15A mice and contrasted their results with phenotypes exhibiting CDKL5 deficiency [7]. Intriguingly, specific changes in some behavioral tests were observed with sex/genotype differences. Specifically, male HOM S15A mice showed impaired functions in locomotion and voluntary wheel tests, but did not display overactivity or anxiety in open field test, as observed in CDKL5 deficient mice. S15A female mice showed impairments in sociability. For both sexes, deficiencies in memory formation and retention were found in the fear conditioning test. With regards to epileptic activities, the authors did not observe spontaneous seizure activities in HOM S15A mice. However, through using low-dose kainic acid injections, female S15A mice exhibited a reduced threshold to generalized seizures along with an increase in ECoG activities. Additionally, the authors provided some clues which may explain the high epileptic seizure susceptibility in CDKL5 deficient phenotypes, and increased Cav2.3 functions and decreased pS15 levels were observed in 22-week-old mice.

In summary, the authors presented substantial experimental evidence, both in vitro and vivo, indicating that deletion of *CDKL5* leads to a decrease in Cav2.3 S15 phosphorylation. This, in turn, causes a delay in Cav2.3 inactivation and an increase in its gating modulation, ultimately resulting in behavioral deficits and an increased susceptibility to epilepsy. Although the exact mechanism of neuronal hyperexcitability in CDD exists as an unillustrated issue, this study offers a unique insight into the role of Cav2.3. These solid findings may further support the therapeutic potential of targeting Cav2.3 for the treatment of CDD-related intractable epilepsy. Based on the current and other related studies, it would be interesting and essential to further explore this field. Firstly, testing the efficacy of current calcium channel blockers in laboratory settings would be worth considering. Additionally, as discussed by the authors, the HOM S15A mice did not exhibit identical behavioral phenotypes as *CDKL5*^*−/−*^ mice. Given that another recent study has proposed the involvement of TrkB signaling related synaptic alteration in spontaneous seizures caused by loss-of-function in the *CDKL5 *gene[8], it is quite possible that besides Cav2.3, other substrates of CDKL5 are also crucial for the pathological conditions in CDD. Therefore, further systematic investigations are needed to understand the process from CDKL5 deficiency to behavioral abnormities. Particularly, considering that epilepsy is gradually accepted as a dysfunction of crucial brain circuits composed of different types of neurons and neuroglia [9, 10], future efforts should focus on exploring the circuitry and cell type-specific role of CDKL5.

## Conclusion

In conclusion, this study provided fresh insight into the epileptic encephalopathies linked to CDKL5 and highlighted Cav2.3 as a possible target for therapeutic interventions. Future efforts should focus on exploring the process from CDKL5 deficiency to behavioral abnormalities and investigating the circuitry and cell type-specific role of CDKL5.

## Data Availability

Not applicable.

## Abbreviations

CDKL5: Cyclin dependent kinase like-5
CDD: CDKL5 deficiency disorder
DEE: Developmental and epileptic encephalopathies
HOM S15A: Homozygous S15A mice
LOF: Loss-of-function
M1: Muscarinic receptor type 1
M3: Muscarinic receptor type 3
